# Early Growth Response Factor 1 in Aging Hematopoietic Stem Cells and Leukemia

**DOI:** 10.3389/fcell.2022.925761

**Published:** 2022-07-18

**Authors:** Rohan Kulkarni

**Affiliations:** The Ohio State University Comprehensive Cancer Center, Columbus, OH, United States

**Keywords:** HSC rejuvenation, leukemia, HSC activation, aged HSCs, hematopoitic stem cells, early growth response 1 (EGR1)

## Abstract

Aging is associated with various hematological disorders and a higher risk of myeloproliferative disorders. An aged hematopoietic system can be characterized by decreased immune function and increased myeloid cell production. Hematopoietic stem cells (HSCs) regulate the production of blood cells throughout life. The self-renewal and regenerative potential of HSCs determine the quality and quantity of the peripheral blood cells. External signals from the microenvironment under different conditions determine the fate of the HSCs to proliferate, self-renew, differentiate, or remain quiescent. HSCs respond impromptu to a vast array of extracellular signaling cascades such as cytokines, growth factors, or nutrients, which are crucial in the regulation of HSCs. Early growth response factor 1 (EGR1) is one of the key transcription factors controlling HSC proliferation and their localization in the bone marrow (BM) niche. Downregulation of *Egr1* activates and recruits HSCs for their proliferation and differentiation to produce mature blood cells. Increased expression of *Egr1* is implicated in immuno-aging of HSCs. However, dysregulation of *Egr1* is associated with hematological malignancies such as acute myeloid leukemia (AML), acute lymphoblastic leukemia (ALL), and chronic myelogenous leukemia (CML). Here, we summarize the current understanding of the role of EGR1 in the regulation of HSC functionality and the manifestation of leukemia. We also discuss the alternative strategies to rejuvenate the aged HSCs by targeting EGR1 in different settings.

## Introduction

Hematopoietic stem cells (HSCs) maintain the peripheral blood pool by regulating the delicate balance between self-renewal, proliferation, and differentiation. Quiescent—long-term HSCs reside at the apex position of the differentiation pyramid, thus holding prime importance in the development of the entire hematopoietic system. These cells give rise to the activated—short-term HSCs which have higher proliferative and differentiation capability. Further, the short-term activated HSCs form the multipotent as well as the lineage-committed progenitor cells that eventually differentiate into various types of functional and mature blood cells (Orkin and Zon, 2008).

HSCs respond to various intrinsic and extrinsic signals and regulate their fate decisions accordingly (Ross and Li, 2006; Zon, 2008). Aging brings about drastic changes in the HSC microenvironment (Latchney and Calvi, 2017; Comazzetto et al., 2021), thereby altering the normal BM niche - HSC inter-communication and affecting the HSC functionality. Moreover, the aged stem cells may lose their ability to sense nutrients and respond to microenvironmental stimuli (López-Otín et al., 2013).

Extrinsic stimuli largely govern the cellular responses and determine cell fate. The quest to understand the immediate effector molecules responding to a wide array of mitogens led to the discovery of a zinc finger transcription factor- early growth response factor 1 (EGR1) (Sukhatme et al., 1987). *Egr1* expression is induced during cell growth, differentiation, and cellular depolarization in a similar manner as governed by c-myc and c-fos and it is co-regulated with c-fos (Sukhatme et al., 1988; Tsai-Morris et al., 1988; Seyfert et al., 1990). The potential targets of EGR1 are tumor necrosis factors (TNFs), interleukin 2 (IL-2), CD44, and Intercellular adhesion molecule 1 (ICAM-1) (McMahon and Monroe, 1996). EGR1 regulates the transcription of more than 30 genes involved in growth, development, and differentiation. It has been also reported to have a transformation suppression activity. The EGR1 molecule has three zinc finger motifs at the C-terminal end, which confer the ability to bind DNA and various promotors having GC rich elements GCEs, (Liu et al., 1996). Thus, EGR1 regulates the expression of various growth factors upon immediate induction by the mitogenic stimulus. Similarly, EGR1 is also reported to have TGF-ß mediated growth and transformation suppression activity (Liu et al., 1996). As EGR1 is prominently involved in establishing early cell proliferative responses to the extrinsic signaling molecules, understanding its role in the regulation of HSCs and hematopoietic disorders becomes essential.

## EGR1 in Hematopoiesis

Earlier studies with the interleukin 3 (IL-3) dependent hematopoietic precursor 32Dcl3 cells, showed that the ectopic overexpression of *Egr1* in them blocked the granulocyte differentiation despite the presence of granulocyte colony-stimulating factor (G-CSF). These cells initiate their terminal differentiation solely into macrophage lineage in presence of granulocyte-macrophage colony-stimulating factor (GM-CSF) (Krishnaraju et al., 1995). Further, the same group studied the effect of overexpression of *Egr1* in stem cell-enriched bone marrow (BM) cells and myeloid enriched BM cells. The study confirmed the shift in the differentiation pattern of stem cell-enriched BM cells from granulocyte or erythroid lineage towards the macrophage lineage upon enhanced EGR1 activity. Moreover, even the myeloid enriched BM cells population in the study differentiated toward macrophage lineage despite the presence of erythropoietin (EPO) and IL-3 (Krishnaraju et al., 2001). Hematopoietic progenitors overexpressing *Egr1* failed to establish the BM engraftment upon transplantation in lethally irradiated mice. This resulted in reduced survival of the recipients due to excessive differentiation towards the macrophage lineage (Krishnaraju et al., 2001). The antisense oligomers targeting *Egr1* RNA block the macrophage differentiation while its constitutive expression specifically induced macrophage differentiation in myeloblastic cell lines. Thus, the *Egr1* gene was identified to positively regulate myeloid differentiation. (Nguyen et al., 1993).

Mice with disrupted *Egr1* alleles show no prominent differences in macrophage differentiation or various macrophage compartments when compared with the wild-type mice. Under normal culture conditions, the BM cells from these mice have similar differentiation responses to various cytokines required for inducing myeloid differentiation, such as macrophage colony-stimulating factor (M-CSF), GM-CSF, or G-CSF. The *Egr1* ^−/−^ macrophages demonstrate unaltered activation potential and have a comparable expression of major histocompatibility complex molecules class-II (MHC class-II). These mice have normal pathogen responses with a comparable life span to that of wild-type mice (Lee et al., 1996). This evidence suggests that *Egr1* expression can induce a differentiation bias towards the macrophage lineage, but it is dispensable for normal hematopoiesis and differentiation. It advocates the compensatory effect of structurally similar Egr family members such as *Egr2*, *Egr3*, and *Egr4* in absence of *Egr1* (Beckmann and Wilce, 1997; Tourtellote et al., 2000). *Egr1* and *Egr2* are redundant in promoting macrophage differentiation and neutrophil repression. However, simultaneous loss of both, *Egr1* and *Egr2* is embryonically lethal. Interestingly, *Egr1* ^−/−^ *Egr2* ^−/+^ compound mutant mice are viable but show reduced weight and size. *Egr1* ^−/−^ *Egr2* ^−/+^ compound mutant mice also show a reduced number of BM HSCs along with a reduced number of functionalities of macrophage progenitors evident from the decreased size of colony-forming units- macrophage (CFU-M) in methylcellulose assay. Their differentiation into macrophages upon GM-CSF or M-CSF stimulation is also reduced, which underscores the role of *Egr1* and *Egr2* in the lineage commitment of progenitor cells. EGR1 and EGR2 do so by counteracting with the neutrophil priming regulator, GIF-1, and positive regulation of macrophage-specific gene expression (Laslo et al., 2006).

In B-lymphocytes, EGR1 is known to regulate the expression of the homing and migration molecule- CD44. Increased *Cd44* expression seen after increased nuclear *Egr1* transcription is a result of binding of newly synthesized EGR to the *Cd44* promotor ∼300 bps before the transcription start point (Maltzman et al., 1996). Similarly, overexpression of *Egr1* in murine B-cell line K46 leads to an increase in *Cd44* expression and downregulation of *Fas/Apo-1* and *Cd23* molecules which reduce the apoptosis mediated cell death (Dinkel et al., 1997). Studies with transgenic mice overexpressing *Egr1* confirm that the EGR1 promotes B and T cells maturation as evident by an increase in the number of mature cells and a concomitant decrease in cells at the immature stage (Miyazaki, 1997; Dinkel et al., 1998). Further findings in *Egr1* ^−/−^ mice validate that Egr1 is dispensable for B cell maturation. However, RGR1 is important for the differentiation of B cells into plasma cells (Oh et al., 2015). The role of *Egr1* and *Egr2* expression is implicated in NKT cell differentiation in response to T cell receptor (TCR) signaling (Seiler et al., 2012). Enforced expression of *Egr1* also increases the megakaryocyte differentiation evident by increased expression of megakaryocyte marker CD41a on K562 cells (Cheng et al., 1994). Thus, EGR1 is well established as the regulator of hematopoietic cell differentiation and maturation.

## EGR1 in Aging Hematopoietic Stem Cells

Aging, in general, is characterized by the progressive loss in functionality of various vital organs, tissues, and cells. This cellular and molecular deterioration at the functional level ultimately results in increased vulnerability to death. Stem cell exhaustion increased cellular senescence and altered intercellular communication are the major hallmarks of aging (López-Otín et al., 2013). Aging alters immune system compositions and impairs lymphopoiesis (Linton and Dorshkind, 2004). The altered differentiation capacity of the aged HSCs increases myeloid cell frequencies and decreases lymphoid cell frequencies in the peripheral blood (Geiger and Van Zant, 2002). The frequency of myeloid biased HSCs also increases during aging (Gekas and Graf, 2013). Aged HSCs demonstrate increased quiescence (Beerman et al., 2014), and delayed cell cycle progression (Noda et al., 2009), with an increase in the senescent cell population (Chen, 2004). Quiescent aged HSCs are capable of repairing their accumulated DNA damage upon re-entry into the cell cycle (Beerman et al., 2014). Aging-related changes in HSCs are the implications of intrinsic dysregulations (Mejia-Ramirez, and Florian, 2020) as well as the contributions of an altered HSC microenvironment (Kulkarni et al., 2018; Ho and Méndez-Ferrer, 2020; Kulkarni and Kale, 2020). As these mechanisms have a proven relation to *Egr1* expression, it is of utmost interest to explore its link with HSC aging.

Overexpression of *Egr1* in *Caenorhabditis elegans* promotes longevity and improves stress resistance to heat and ultraviolet light. EGR1 modulates the insulin signaling cascade and eventually increases the lifespan. While decreased EGR1 signaling in *C. elegans* results in the reduced lifespan of these worms. The same study reports that the expression of Egr1 increases with age and protects the organism from aging-related dysfunctions (Zimmerman and Kim, 2014). Another study shows that the deletion of the *Egr1* results in a striking phenotype with complete bypass of senescence and induced immortal cell growth ability with a concomitant decrease in *p53* and *p21* ^Cip1/Waf1^ signaling (Krones-Herzig et al., 2003). This highlights the role of *Egr1* in the regulation of senescence and cell death. EGR1 is also a well-known regulator of aging-related genes (Baron et al., 2005) such as transforming growth factor β1 (*Tgf- β1*) (Valletta et al., 2020), phosphatase and tensin homolog (*Pten*) (Chen et al., 2009), p53 (Dumble et al., 2007). A detailed study with the *Egr1* ^−/−^ mice model validates the significance of EGR1 in HSC proliferation and the HSCs localization in the BM niche. *Egr1* ^−/−^ mice show a significant increase in HSC cycling with about a two-fold increase in the HSC percentages present in the S-G2-M phase. These HSCs also exhibit significant downregulation of *p21* ^Cip1/Waf1^ expression along with an increase in *Cdk4* expression in them. Loss of *Egr1* does not alter the long-term reconstitution potential of the HSCs but results in premature exhaustion of stem cells due to the high proliferation rate upon serial transplantation. G-CSF-induced mobilization of HSCs also downregulates Egr1 expression, signifying its role in HSC localization under a steady state. On the other hand, *Egr1* ^−/−^ mice demonstrate spontaneous mobilization of HSCs from the BM, confirming the importance of EGR1 in HSC localization (Min et al., 2008).

Recently, Desterke et al. analyzed the single-cell transcriptomics of the aged human HSCs. They found that the EGR1 is substantially upregulated in aged Lineage^-^ CD34^-^ CD38^-^ cells. These cells exhibit dysregulation of the cell cycle with a reduction in cell population present in the G2-M phase and a reduction in CCND2 expression during the S phase. Aged HSCs also show induced expression of the other molecules which are generally implicated in the hematopoietic and immune disorders, AP-1, and HSC quiescence regulators such as BTG, JUNB, and NR41A. Aged human HSCs present dysregulation of CDH4/6/D type cyclin complex and CDK6-EGR1 axis, which has an important implication in the activation of quiescent HSCs (Desterke et al., 2021). CDK6 suppresses the transcription of *Egr1* during the recruitment of quiescent HSCs for activation. Loss of *Cdk6* in HSCs induces an increase in the frequency of quiescent HSCs resulting in their failure to repopulate bone marrow after transplantation. Consistent *Egr1* expression despite repeated 5-FU treatment leads to the inability of *Cdk6* ^−/−^ mice to recruit HSCs for activation. This significantly hampers the survival of the mice due to reduced hematopoietic activity under induced stress (Scheicher et al., 2015). Single-cell RNA sequencing analysis and bulk RNA sequencing strategies have demonstrated a notable increase in *EGR1* expression in aged human HSCs (Adelman et al., 2019). Another report on single-cell sequencing for combined analysis of epigenetic, transcriptomic, and functional data identified the effect of *Egr1* expression in fetal HSCs. The report showed a correlation between DNA hypermethylation with decreased expression of *Egr1* transcription network genes including SOCS3, KLF2, and JUNB. These changes are associated with decreased activation of HSCs and the inability to respond to the stimulation (Pelletier et al., 2021). Thes data suggest the importance of EGR1 in HSC cell cycle regulation, activation, localization, and differentiation.

Bone marrow stromal cells (BMSCs) regulate HSC functionality *in-vivo* (Garcia-Garcia et al., 2015) and have been used to support the HSCs for *in-vitro* expansion cultures (Jing et al., 2010). A recent study confirms the role of *Egr1* in BMSC proliferation. *Egr1* expression levels are found to be high in the freshly isolated BMSCs but are rapidly downregulated upon proliferation under culturing conditions. However, *Egr1* expression levels are also seen to be reduced in the colony-forming BM MSCs. Enforced expression of *Egr1* decreases proliferation in stromal cells but upon culturing for HSC expansion, their HSC support potential increases, evident through the elevated number of transplantable HSCs (Li et al., 2020). Netrin-1, expressed on arteriolar endothelial and periarteriolar stromal cells, regulates niche-dependent HSC self-renewal and quiescence through its interaction with Neogenin-1 by triggering *Egr1* expression. The aged niche shows a decline in Netrin-1, which induces compensatory overexpression of Neogenin-1 in HSCs, inducing Egr1 expression further (Renders et al., 2021). These findings suggest that *Egr1* expression during aging is amendable by extrinsic modulations and can be a potential target for HSC rejuvenation therapies.

## Differential Role of EGR1 in Mature and Immature Cell Types

EGR1 is a master transcription factor that can regulate various genes by acting as an activator or a suppressor, depending on distinct cofactors associated with it (Thiel and Cibelli, 2002). Importantly, EGR1 regulates immune cells through its target genes such as *Il-2*, *Cd44*, *Icam*, and *Tnf* (McMahon and Monroe, 1996). In the case of the cancer cells, Wang et al. have reported that high glucose levels induce cell proliferation *via* increased *Tgf- β1* levels, mediated by increased binding of EGR1 to the *Tgf- β1* promoter site (Wang et al., 2015).

While in HSCs, increased TGF- β signaling induces cell hibernation *via* inhibition of cytokine-mediated lipid raft clustering, which is required for re-entering the cell cycle (Yamazaki et al., 2009). *Egr1* promotor also has EGF response elements and serum response elements which induce *Egr1* expression through the EGF-ERK-Elk1 signaling cascade (Gregg and Fraizer, 2011). Though ERK is also known to induce the proliferation of cells, the ERK-dependent feedback mechanism prevents HSC exhaustion during hematopoiesis (Baumgartner et al., 2018). Cyclin D1 transcription during progression through the G1 to S phase of the cell cycle requires MAPK-induced *Egr1* expression and its binding to the promotor (Xiao et al., 2005). On the other hand, maintenance of self-renewal in HSCs requires balanced MAPK signaling (Geest and Coffer, 2009). As discussed earlier, CDK6 acts as the transcriptional suppressor of *Egr1* in HSCs, which is required for their activation (Scheicher et al., 2015). Moreover, *Egr* ^−/−^ HSCs show increased cycling with a significant increase in *Cdk4* expression without any significant change in the cyclin D1 expression levels (Min et al., 2008). These reports suggest that EGR1 can play different roles in mature and stem cell populations depending on the upstream and downstream signaling cascades and regulation of EGR1 expression levels.

## EGR1 in Leukemia

Alongside the role of *Egr1* in normal hematopoiesis discussed in the previous section, its role in mature or leukemic hematopoietic cells has also been studied extensively. Ectopic overexpression of EGR1 in M1 myeloblastic leukemia cells abrogates the G0/G1 block imparted by oncogenic c-Myc or E2F-1 in these cells. Further, EGR1 commits them to terminal differentiation under the effect of extrinsic IL-6 treatment and diminishes the aggression of the M1 myeloblastic leukemic cells (Gibbs et al., 2008). In acute lymphoblastic leukemia (ALL) EGR1 induction *via* suppression of HSP70 induces apoptosis and inhibits cell proliferation, whereas loss of EGR1 in these cells increased the proliferation and reduces apoptosis of ALL cells (Guo et al., 2019). Upregulation of EGR1 in leukemic cells suppresses invasion and migration of the leukemic cell (Liu et al., 2009). Tumor suppressor activity of EGR1 is well proven in Chronic Myelogenous Leukemia (CML) as well (Maifrede et al., 2014). Importantly, the EGR1 gene is located on chromosome 5, in a region that is often found to be deleted in acute myeloid leukemia (AML) cells (Sukhatme et al., 1988). In the AML settings, upregulation of EGR1 has been shown to down-regulate Survivin, an inhibitor of apoptosis, and regulator of cell division, which also sensitizes AML cells to TRAIL-induced apoptosis (Tamm et al., 2006).

In multiple myeloma cells, JUN overexpression induces cell death and growth inhibition by upregulation of the EGR1, which in turn downregulates Survivin and triggers caspase signaling. Interestingly improved outcome for the bortezomib treatment is associated with increased JUN or *Egr1* expression, while JUN or EGR1 knockdown increases the resistance of myeloma cells against bortezomib (Chen et al., 2010). In lymphoblastic leukemia, *miR-181a* can target and downregulate EGR1 which increases the proliferation and survival of leukemic cells leading to negative outcomes (Verduci et al., 2015). EGR1 expression in the tumor microenvironment has been also proven to inhibit tumor growth. Ectopic expression of EGR1 in thymic lymphoma stromal cells downregulates matrix metalloproteinase-9 (MMP-9) expression in them and inhibits the lymphoma growth (Bouchard et al., 2010). Overall, *Egr1* ability to induce terminal differentiation of various mature hematopoietic cells and its tumor-suppressive activity have been explored as potential anti-leukemia therapies.

## Discussion

**FIGURE 1:**
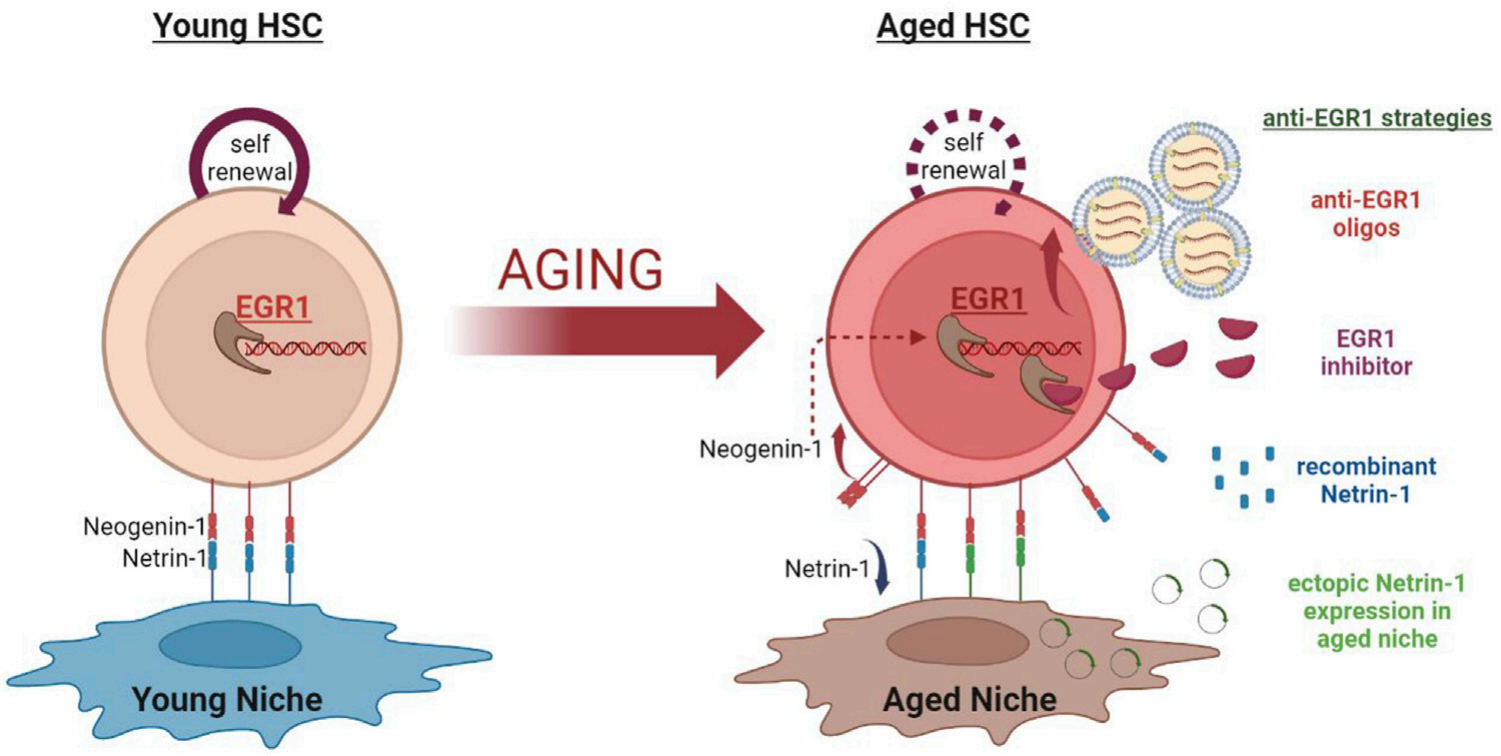
EGR1 targeting strategies for rejuvenation of aged Hematopoietic Stem Cells. The expression of the zinc finger transcription factor - EGR1 is significantly increased in aged HSCs. This is, at least partially, mediated by the aging-associated changes in the BM niche. Increased EGR1 activation in the aged HSCs hampers their self-renewal activity and functions. EGR1 targeting strategies may be used to explore the possibility to rejuvenate aged HSCs. These strategies include - **(A)** Using the vesicle-mediated transfer of anti-Egr1 oligos specifically targeting aged HSCs. **(B)** Developing pharmacological EGR1 inhibitors. **(C)** Treating aged HSCs with recombinant Netrin-1 protein to reduce EGR1 activation in them. **(D)** Ectopic expression of Netrin-1 in aged BM niche cells to rescue Netrin-1 -Neogenin-1 interaction to modulate downstream EGR1 levels in the aged HSCs.

EGR1 being the regulator of HSC quiescence, proliferation, and localization is the key factor in determining HSC function and fate. *Egr1* deletion also inhibits cellular senescence and induces proliferation in senescent cells. Recent evidence suggests dysregulation of EGR1 in the aged HSCs. Aged HSCs have increased quiescence and senescence which results in decreased HSC functionality. In the scenario of being enforced to enter the cell cycle, aged HSCs repair the accumulated DNA damage. Egr1 downregulation might decrease senescence and induce their activation in aged HSCs, in turn improving their functionality. Thus, studying the effect of extrinsic modulation of EGR1 in aged HSCs needs to be investigated for potential strategies for rejuvenation of the aged HSCs. Some of these promising approaches include- niche-mediated modulatory mechanisms, targeted vesicle-mediated antisense oligonucleotide delivery, and the development of EGR1 inhibitors (Figure 1).

## References

[B1] Adelman E. R. Huang H.-T. Roisman A. Olsson A. Colaprico A. Qin T. (2019). Aging Human Hematopoietic Stem Cells Manifest Profound Epigenetic Reprogramming of Enhancers that May Predispose to Leukemia. Cancer Discov. 9 (8), 1080–1101. 10.1158/2159-8290.cd-18-1474 31085557 PMC7080409

[B3] Baron V. Vo C. Mercola D. (2005). A Combination Study of Egr-1 Antisense with Chemotherapeutic Drugs in Human Prostate Cancer Cells, 65. United States: American Association for Cancer Research, 140.

[B4] Baumgartner C. Toifl S. Farlik M. Halbritter F. Scheicher R. Fischer I. (2018). An ERK-dependent Feedback Mechanism Prevents Hematopoietic Stem Cell Exhaustion. Cell stem Cell 22 (6), 879–892. 10.1016/j.stem.2018.05.003 29804890 PMC5988582

[B5] Beckmann A. M. Wilce P. A. (1997). Egr Transcription Factors in the Nervous System. Neurochem. Int. 31 (4), 477–510. 10.1016/s0197-0186(96)00136-2 9307998

[B6] Beerman I. Seita J. Inlay M. A. Weissman I. L. Rossi D. J. (2014). Quiescent Hematopoietic Stem Cells Accumulate DNA Damage during Aging that Is Repaired upon Entry into Cell Cycle. Cell stem Cell 15 (1), 37–50. 10.1016/j.stem.2014.04.016 24813857 PMC4082747

[B7] Bouchard F. Bélanger S. D. Biron-Pain K. St-Pierre Y. (2010). EGR-1 Activation by EGF Inhibits MMP-9 Expression and Lymphoma Growth. Blood, J. Am. Soc. Hematol. 116 (5), 759–766. 10.1182/blood-2009-12-257030 20472833

[B8] Chen C. Liu Y. Liu Y. Zheng P. (2009). mTOR Regulation and Therapeutic Rejuvenation of Aging Hematopoietic Stem Cells. Sci. Signal 2 (98), ra75. 10.1126/scisignal.2000559 19934433 PMC4020596

[B9] Chen J. (2004). Senescence and Functional Failure in Hematopoietic Stem Cells. Exp. Hematol. 32 (11), 1025–1032. 10.1016/j.exphem.2004.08.001 15539079

[B10] Chen L. Wang S. Zhou Y. Wu X. Entin I. Epstein J. (2010). Identification of Early Growth Response Protein 1 (EGR-1) as a Novel Target for JUN-Induced Apoptosis in Multiple Myeloma. Blood, J. Am. Soc. Hematol. 115 (1), 61–70. 10.1182/blood-2009-03-210526 PMC2803692 19837979

[B11] Cheng T. Wang Y. Dai W. (1994). Transcription Factor Egr-1 Is Involved in Phorbol 12-myristate 13-Acetate-Induced Megakaryocytic Differentiation of K562 Cells. J. Biol. Chem. 269 (49), 30848–30853. 10.1016/s0021-9258(18)47359-0 7983016

[B12] Comazzetto S. Shen B. Morrison S. J. (2021). Niches that Regulate Stem Cells and Hematopoiesis in Adult Bone Marrow. Dev. Cell 56 (13), 1848–1860. 10.1016/j.devcel.2021.05.018 34146467 PMC8282762

[B62] Desterke C. Bennaceur-Griscelli A. Turhan A. G. (2021). EGR1 Dysregulation Defines an Inflammatory and Leukemic Program in Cell Trajectory of Human-Aged Hematopoietic Stem Cells (HSC). Stem cell Res. Ther. 12 (1), 1–20. 34294125 10.1186/s13287-021-02498-0 PMC8296523

[B13] Dinkel A. Aicher W. K. Haas C. Zipfel P. F. Peter H. H. Eibel H. (1997). Transcription Factor Egr-1 Activity Down-Regulates Fas and CD23 Expression in B Cells. J. Immunol. 159 (6), 2678–2684. 9300687

[B14] Dinkel A. Warnatz K. Ledermann B. Rolink A. Zipfel P. F. Bürki K. (1998). The Transcription Factor Early Growth Response 1 (Egr-1) Advances Differentiation of Pre-B and Immature B Cells. J. Exp. Med. 188 (12), 2215–2224. 10.1084/jem.188.12.2215 9858508 PMC2212439

[B15] Dumble M. Moore L. Chambers S. M. Geiger H. Van Zant G. Goodell M. A. (2007). The Impact of Altered P53 Dosage on Hematopoietic Stem Cell Dynamics during Aging. Blood 109 (4), 1736–1742. 10.1182/blood-2006-03-010413 17032926 PMC1794064

[B16] García-García A. de Castillejo C. L. F. Méndez-Ferrer S. (2015). BMSCs and Hematopoiesis. Immunol. Lett. 168 (2), 129–135. 10.1016/j.imlet.2015.06.020 26192443

[B17] Geest C. R. Coffer P. J. (2009). MAPK Signaling Pathways in the Regulation of Hematopoiesis. J. Leukoc. Biol. 86 (2), 237–250. 10.1189/jlb.0209097 19498045

[B18] Geiger H. Van Zant G. (2002). The Aging of Lympho-Hematopoietic Stem Cells. Nat. Immunol. 3 (4), 329–333. 10.1038/ni0402-329 11919569

[B19] Gekas C. Graf T. (2013). CD41 Expression Marks Myeloid-Biased Adult Hematopoietic Stem Cells and Increases with Age. J. Am. Soc. Hematol. 121 (22), 4463–4472. 10.1182/blood-2012-09-457929 23564910

[B20] Gibbs J. D. Liebermann D. A. Hoffman B. (2008). Egr-1 Abrogates the E2F-1 Block in Terminal Myeloid Differentiation and Suppresses Leukemia. Oncogene 27 (1), 98–106. 10.1038/sj.onc.1210627 17599039

[B21] Gregg J. Fraizer G. (2011). Transcriptional Regulation of EGR1 by EGF and the ERK Signaling Pathway in Prostate Cancer Cells. Genes & cancer 2 (9), 900–909. 10.1177/1947601911431885 22593802 PMC3352154

[B22] Guo D. Zhang A. Huang J. Suo M. Zhong Y. Liang Y. (2019). Suppression of HSP70 Inhibits the Development of Acute Lymphoblastic Leukemia via TAK1/Egr-1. Biomed. Pharmacother. 119, 109399. 10.1016/j.biopha.2019.109399 31521893

[B23] Ho Y.-H. Méndez-Ferrer S. (2020). Microenvironmental Contributions to Hematopoietic Stem Cell Aging. Haematologica 105 (1), 38–46. 10.3324/haematol.2018.211334 31806690 PMC6939521

[B24] Jing D. Fonseca A. V. Alakel N. Fierro F. A. Muller K. Bornhauser M. (2010). Hematopoietic Stem Cells in Co-culture with Mesenchymal Stromal Cells - Modeling the Niche Compartments *In Vitro* . haematologica 95 (4), 542–550. 10.3324/haematol.2009.010736 20145267 PMC2857183

[B25] Krones-Herzig A. Adamson E. Mercola D. (2003). Early Growth Response 1 Protein, an Upstream Gatekeeper of the P53 Tumor Suppressor, Controls Replicative Senescence. Proc. Natl. Acad. Sci. U.S.A. 100 (6), 3233–3238. 10.1073/pnas.2628034100 12629205 PMC152275

[B64] Krishnaraju K. Nguyen H. Q. Liebermann D. A. Hoffman B. (1995). The Zinc Finger Transcription Factor Egr-1 Potentiates Macrophage Differentiation of Hematopoietic Cells. Molecul. Cellul. Biol. 15 (10), 5499–5507. 10.1128/mcb.15.10.5499 PMC230800 7565701

[B65] Krishnaraju K. Hoffman B. Liebermann D. A. (2001). Early Growth Response Gene 1 Stimulates Development of Hematopoietic Progenitor Cells Along the Macrophage Lineage at the Expense of the Granulocyte and Erythroid Lineages. Blood, J. Amer. Soc. Hematol. 97 (5), 1298–1305. 10.1182/blood.v97.5.1298 11222373

[B26] Kulkarni R. Bajaj M. Ghode S. Jalnapurkar S. Limaye L. Kale V. P. (2018). Intercellular Transfer of Microvesicles from Young Mesenchymal Stromal Cells Rejuvenates Aged Murine Hematopoietic Stem Cells. Stem Cells 36 (3), 420–433. 10.1002/stem.2756 29230885

[B27] Kulkarni R. Kale V. (2020). Physiological Cues Involved in the Regulation of Adhesion Mechanisms in Hematopoietic Stem Cell Fate Decision. Front. Cell Dev. Biol. 8, 611. 10.3389/fcell.2020.00611 32754597 PMC7366553

[B28] Laslo P. Spooner C. J. Warmflash A. Lancki D. W. Lee H.-J. Sciammas R. (2006). Multilineage Transcriptional Priming and Determination of Alternate Hematopoietic Cell Fates. Cell 126 (4), 755–766. 10.1016/j.cell.2006.06.052 16923394

[B29] Latchney S. E. Calvi L. M. (2017). The Aging Hematopoietic Stem Cell Niche: Phenotypic and Functional Changes and Mechanisms that Contribute to Hematopoietic Aging. Seminors Hematol 54, 25–32. WB Saunders. 10.1053/j.seminhematol.2016.10.001 PMC5244432 28088984

[B30] Lee S. L. Wang Y. Milbrandt J. (1996). Unimpaired Macrophage Differentiation and Activation in Mice Lacking the Zinc Finger Transplantation Factor NGFI-A (EGR1). Mol. Cell Biol. 16 (8), 4566–4572. 10.1128/mcb.16.8.4566 8754857 PMC231455

[B31] Li H. Lim H.-C. Zacharaki D. Xian X. Kenswil K. J. G. Bräunig S. (2020). Early Growth Response 1 Regulates Hematopoietic Support and Proliferation in Human Primary Bone Marrow Stromal Cells. haematologica 105 (5), 1206–1215. 10.3324/haematol.2019.216648 31371413 PMC7193482

[B32] Linton P. J. Dorshkind K. (2004). Age-related Changes in Lymphocyte Development and Function. Nat. Immunol. 5 (2), 133–139. 10.1038/ni1033 14749784

[B33] Liu C. Adamson E. Mercola D. (1996). Transcription Factor EGR-1 Suppresses the Growth and Transformation of Human HT-1080 Fibrosarcoma Cells by Induction of Transforming Growth Factor Beta 1. Proc. Natl. Acad. Sci. U.S.A. 93 (21), 11831–11836. 10.1073/pnas.93.21.11831 8876223 PMC38144

[B34] Liu P. Li J. Lu H. Xu B. (2009). Thalidomide Inhibits Leukemia Cell Invasion and Migration by Upregulation of Early Growth Response Gene 1. Leukemia lymphoma 50 (1), 109–113. 10.1080/10428190802588352 19152168

[B35] López-Otín C. Blasco M. A. Partridge L. Serrano M. Kroemer G. (2013). The Hallmarks of Aging. Cell 153 (6), 1194–1217. 10.1016/j.cell.2013.05.039 23746838 PMC3836174

[B36] Maifrede S. Liebermann D. Hoffman B. (2014). Egr-1, a Stress Response Transcription Factor and Myeloid Differentiation Primary Response Gene, Behaves as Tumor Suppressor in CML. Blood 124 (21), 2211. 10.1182/blood.v124.21.2211.2211

[B37] Maltzman J. S. Carman J. A. Monroe J. G. (1996). Role of EGR1 in Regulation of Stimulus-dependent CD44 Transcription in B Lymphocytes. Mol. Cell Biol. 16 (5), 2283–2294. 10.1128/mcb.16.5.2283 8628295 PMC231216

[B38] McMahon S. B. Monroe J. G. (1996). The Role of Early Growth Response Gene 1 (Egr -1) in Regulation of the Immune Response. J. Leukoc. Biol. 60 (2), 159–166. 10.1002/jlb.60.2.159 8773576

[B39] Mejia-Ramirez E. Florian M. C. (2020). Understanding Intrinsic Hematopoietic Stem Cell Aging. haematologica 105 (1), 22–37. 10.3324/haematol.2018.211342 31806687 PMC6939535

[B40] Min I. M. Pietramaggiori G. Kim F. S. Passegué E. Stevenson K. E. Wagers A. J. (2008). The Transcription Factor EGR1 Controls Both the Proliferation and Localization of Hematopoietic Stem Cells. Cell stem Cell 2 (4), 380–391. 10.1016/j.stem.2008.01.015 18397757

[B41] Miyazaki T. (1997). Two Distinct Steps during Thymocyte Maturation from CD4−CD8− to CD4+CD8+ Distinguished in the Early Growth Response (Egr)-1 Transgenic Mice with a Recombinase-Activating Gene-Deficient Background. J. Exp. Med. 186 (6), 877–885. 10.1084/jem.186.6.877 9294142 PMC2199048

[B42] Nguyen H. Q. Hoffman-Liebermann B. Liebermann D. A. (1993). The Zinc Finger Transcription Factor Egr-1 Is Essential for and Restricts Differentiation along the Macrophage Lineage. Cell 72 (2), 197–209. 10.1016/0092-8674(93)90660-i 7678779

[B63] Noda S. Ichikawa H. Miyoshi H. (2009). Hematopoietic Stem Cell Aging is Associated With Functional Decline and Delayed Cell Cycle Progression. Biochem. Biophys. Res. Communicat. 383 (2), 210–215. 10.1016/j.bbrc.2009.03.153 19345668

[B43] Oh Y.-K. Jang E. Paik D.-J. Youn J. (2015). Early Growth Response-1 Plays a Non-redundant Role in the Differentiation of B Cells into Plasma Cells. Immune Netw. 15 (3), 161–166. 10.4110/in.2015.15.3.161 26140048 PMC4486779

[B44] Orkin S. H. Zon L. I. (2008). Hematopoiesis: an Evolving Paradigm for Stem Cell Biology. Cell 132 (4), 631–644. 10.1016/j.cell.2008.01.025 18295580 PMC2628169

[B45] Pelletier A. Carrier A. Zhao Y. M. Canouil M. Derhourhi M. Durand E. (2021). Excessive Fetal Growth Affects HSC Quiescence Maintenance through Epigenetic Programming of EGR1 Transcriptional Network. NY: bioRxiv.

[B66] Renders S. Svendsen A. F. Panten J. Rama N. Maryanovich M. Sommerkamp P. (2021). Niche Derived Netrin-1 Regulates Hematopoietic Stem Cell Dormancy via its Receptor Neogenin-1. Nat. communicat. 12 (1), 1–15. 10.1038/s41467-020-20801-0 PMC7840807 33504783

[B46] Ross J. Li L. (2006). Recent Advances in Understanding Extrinsic Control of Hematopoietic Stem Cell Fate. Curr. Opin. Hematol. 13 (4), 237–242. 10.1097/01.moh.0000231420.92782.8f 16755219

[B47] Scheicher R. Hoelbl-Kovacic A. Bellutti F. Tigan A.-S. Prchal-Murphy M. Heller G. (2015). CDK6 as a Key Regulator of Hematopoietic and Leukemic Stem Cell Activation. J. Am. Soc. Hematol. 125 (1), 90–101. 10.1182/blood-2014-06-584417 PMC4281832 25342715

[B48] Seiler M. P. Mathew R. Liszewski M. K. Spooner C. J. Barr K. Meng F. (2012). Elevated and Sustained Expression of the Transcription Factors Egr1 and Egr2 Controls NKT Lineage Differentiation in Response to TCR Signaling. Nat. Immunol. 13 (3), 264–271. 10.1038/ni.2230 22306690 PMC3288314

[B49] Seyfert V. L. McMahon S. Glenn W. Cao X. M. Sukhatme V. P. Monroe J. G. (1990). Egr-1 Expression in Surface Ig-Mediated B Cell Activation. Kinetics and Association with Protein Kinase C Activation. J. Immunol. 145 (11), 3647–3653. 2246508

[B50] Sukhatme V. P. Kartha S. Toback F. G. Taub R. Hoover R. G. Tsai-Morris C. H. (1987). A Novel Early Growth Response Gene Rapidly Induced by Fibroblast, Epithelial Cell and Lymphocyte Mitogens. Oncogene Res. 1 (4), 343–355. 3130602

[B51] Sukhatme V. P. Cao X. Chang L. C. Tsai-Morris C.-H. Stamenkovich D. Ferreira P. C. P. (1988). A Zinc Finger-Encoding Gene Coregulated with C-Fos during Growth and Differentiation, and after Cellular Depolarization. Cell 53 (1), 37–43. 10.1016/0092-8674(88)90485-0 3127059

[B52] Tamm I. Schmelz K. Dörken B. Wagner M. (2006). Survivin Is Negatively Regulated by Early Growth Response (Egr)-1 Transcription Factor in Acute Myeloid Leukemia. Blood 108 (11), 1943. 10.1182/blood.v108.11.1943.1943

[B53] Thiel G. Cibelli G. (2002). Regulation of Life and Death by the Zinc Finger Transcription Factor Egr-1. J. Cell. Physiol. 193 (3), 287–292. 10.1002/jcp.10178 12384981

[B67] Tourtellotte W. G. Nagarajan R. Bartke A. Milbrandt J. (2000). Functional Compensation by Egr4 in Egr1-Dependent Luteinizing Hormone Regulation and Leydig Cell Steroidogenesis. Mole. Cellul. Biol. 20 (14), 5261–5268. 10.1128/mcb.20.14.5261-5268.2000 PMC85975 10866682

[B54] Tsai-Morris C.-H. Cao X. Sukhatme V. P. (1988). 5' Flanking Sequence and Genomic Structure of Egr-1, a Murine Mitogen Inducible Zinc Finger Encoding Gene. Nucleic acids Res. 16 (18), 8835–8846. 10.1093/nar/16.18.8835 3140220 PMC338638

[B55] Valletta S. Thomas A. Meng Y. Ren X. Drissen R. Sengül H. (2020). Micro-environmental Sensing by Bone Marrow Stroma Identifies IL-6 and TGFβ1 as Regulators of Hematopoietic Ageing. Nat. Commun. 11 (1), 4075–4113. 10.1038/s41467-020-17942-7 32796847 PMC7427787

[B56] Verduci L. Azzalin G. Gioiosa S. Carissimi C. Laudadio I. Fulci V. (2015). microRNA-181a Enhances Cell Proliferation in Acute Lymphoblastic Leukemia by Targeting EGR1. Leukemia Res. 39 (4), 479–485. 10.1016/j.leukres.2015.01.010 25740602

[B57] Wang D. Guan M.-P. Zheng Z.-J. Li W.-Q. Lyv F.-P. Pang R.-Y. (2015). Transcription Factor Egr1 Is Involved in High Glucose-Induced Proliferation and Fibrosis in Rat Glomerular Mesangial Cells. Cell Physiol. Biochem. 36 (6), 2093–2107. 10.1159/000430177 26279418

[B58] Xiao D. Chinnappan D. Pestell R. Albanese C. Weber H. C. (2005). Bombesin Regulates Cyclin D1 Expression through the Early Growth Response Protein Egr-1 in Prostate Cancer Cells. Cancer Res. 65 (21), 9934–9942. 10.1158/0008-5472.can-05-1830 16267018

[B59] Yamazaki S. Iwama A. Takayanagi S.-i. Eto K. Ema H. Nakauchi H. (2009). TGF-β as a Candidate Bone Marrow Niche Signal to Induce Hematopoietic Stem Cell Hibernation. Blood, J. Am. Soc. Hematol. 113 (6), 1250–1256. 10.1182/blood-2008-04-146480 18945958

[B60] Zimmerman S. M. Kim S. K. (2014). TheGATAtranscription factor/MTA‐1 Homologegr‐1promotes Longevity and Stress Resistance inCaenorhabditis Elegans. Aging Cell 13 (2), 329–339. 10.1111/acel.12179 24304470 PMC4331783

[B61] Zon L. I. (2008). Intrinsic and Extrinsic Control of Haematopoietic Stem-Cell Self-Renewal. Nature 453 (7193), 306–313. 10.1038/nature07038 18480811

